# Evaluation Method for Quality Risks of Safety in Prefabricated Building Construction Using SEM–SDM Approach

**DOI:** 10.3390/ijerph19095180

**Published:** 2022-04-24

**Authors:** Xiaojuan Li, Chen Wang, Mukhtar A. Kassem, Hamed H. Alhajlah, Samuel Bimenyimana

**Affiliations:** 1 College of Transportation and Civil Engineering, Fujian Agriculture and Forestry University, Fuzhou 350108, China; xiaojuanli@fafu.edu.cn; 2 College of Civil Engineering, Huaqiao University, Xiamen 361021, China; s0785213122@gmail.com; 3 Department of Quantity Surveying, Faculty of Built Environment & Surveying, University of Technology Malaysia, Johor Bahru 81310, Malaysia; 4 Civil Engineering Department, Faculty of Engineering, University of Birmingham, Edgbaston, Birmingham B15 2TT, West Midlands, UK; hxa580@alumni.bham.ac.uk

**Keywords:** prefabricated buildings, structural equation modelling, system dynamic model, risk evaluation, quality and safety

## Abstract

The construction of prefabricated buildings is an effective and efficient approach to improving construction processes and productivity. However, there are practical problems in this approach, such as listing, safety risk levels, and quality control. This study aims to develop a systematic approach for determining the key factors affecting prefabricated building projects’ quality and safety risk and assessing this risk. Based on the literature review, a structured questionnaire was distributed to 408 China-based construction organizations. Considering the factors of safety risk evaluation systems for construction, the safety risk model of prefabricated buildings is established and combined with structural equation modeling (SEM) and a system dynamic model (SDM). A detailed case study was conducted to verify the empirical findings. The results show that pre-construction, during-construction, and after-construction significantly influence the quality risk (from high to low). The final comprehensive score is 92.71, indicating that the construction safety of the residential building is generally controllable and the quality is guaranteed. Furthermore, the investment risk of such projects can be assessed using SEM and SDM. This study contributes to the literature by considering quality-risk-influencing factors in this field. Furthermore, the findings provide an understanding of implementation and quality risk control for prefabricated building projects and provide valuable information to departments in charge of improving the safety risk performance of such projects.

## 1. Introduction

The construction and production methods of traditional buildings are backward, and the quality of the labor force is not yet up to an acceptable standard and cannot meet the requirements of the new era for the construction industry. Simple traditional construction methods that waste resources pollute the environment and are inefficient, and they cannot meet the development requirements of the construction industry [1]. Traditional construction models also affect ecology, cities, and economic structures. Therefore, is the need is urgent to transform and improve the traditional construction mode. A prefabricated building formation is a building that must be standardized and designed to meet the use function of general-purpose components produced by the prefabrication plant at the construction site [2]. There are high expectations for developing prefabricated buildings at the national, provincial, and municipal levels [3]. The construction of a prefabricated building is an integration process, taking industrial production as the integration core and achieving the integration of structure, maintenance, interior decoration, equipment, pipelines in engineering, integration of planning, design, production, and construction participants [4]. Prefabricated buildings are standardized and industrialized and transported to the site for hoisting and splicing through special equipment [5]. Industrialized production methods have extensively promoted the process of industrialization of buildings [6]. The characteristics of prefabricated buildings are supposed to be highly efficient, energy-saving, and protective of the environment, with optimized production structures and strong professionalism [7,8]. With the rapid development of prefabricated buildings, the demand for safety quality management in the construction industry has increased rapidly. The Central Committee of the Communist Party of China proposed that prefabricated buildings will account for more than 50 percent of new buildings by 2025 [9]. However, the construction process of prefabricated buildings is more complex than that of ordinary buildings, and the safety quality management of prefabricated buildings is still in its infancy. Therefore, such quality and safety management must implement an efficient and effective management assessment model to improve the production status of prefabricated buildings.

The quality and safety problems in the development of prefabricated buildings are very prominent according to [10], and it is essential to strengthen quality and safety management. Moreover, methods for evaluating the safety risk of prefabricated buildings are lacking in construction enterprises [11]. Additionally, research on evaluation methods has received little attention in previous studies [12,13]. From a systematic literature review, existing studies related to the safety risk have mainly focused on the fundamental theories, models, applications, benefits, and integration with other ideas, such as sustainable development theory, green development theory, and building information modeling technology. To provide enterprises with a theoretical framework for safety risk assessment and focus more attention on research-on-research methods, the paper aims to put forward a method to analyze the influences on quality risk and assess the safety risk of prefabricated building projects. At present, scholars have used data-driven methods such as SEM, SDM, and interpretative structural modeling (ISM) to study the environmental risks of subway construction, railway transportation system, green building construction, and other projects [14,15,16,17]. They have successfully developed a risk assessment system with practical value to identify potential risk factors leading to unsafe accidents, and then take targeted measures to avoid risks and improve the success rate of similar projects. In order to accurately and objectively evaluate the safety risk of prefabricated building projects, SEM and SDM are used to estimate the safety risk level of the prefabricated building construction stage, and an empirical study is used to explain the safety risk factors and determine the trend of safety risk assessment. The findings contribute to the existing knowledge of quality risk factors by focusing on prefabricated building projects. Furthermore, this study can provide theoretical support to provide insights for industry practitioners who seek to adopt a safety risk assessment method.

The safety management process of prefabricated buildings is dynamic. Shewhart puts forward the famous PDCA cycle management principles, namely P (Plan) plan, D (Do) execution, C (Check) inspection, and A (Act) processing [18]. First, the risks list uses the assessment method to determine the risk level of each quality and safety factor, and the solution shows the risk level of each factor [19]. Then, the implementation of the project and the effectiveness of risk management were checked according to the implementation plan. Finally, the risk plan was adjusted after processing the inspection results. Therefore, building safety management is dynamic management. In the prefabricated construction project, the early planning stage is long. Although the relative implementation time of the last project is short, the quality risk control point does not decrease, but increases. Therefore, it is necessary to adjust construction plans to achieve optimal project status on time.

Based on the research of many scholars [20,21,22,23], complex risk factors run through the whole life cycle of the project, which consists of three stages: design, production and installation. Including but not limited to hidden dangers in the connection quality of component nodes, imperfect component transportation and loading and unloading plans, deviations in component installation positions, and immature component modularization technology. The safety management of prefabricated construction projects covers the stage from producing prefabricated components in the prefabricated plant to complete protection. By analyzing the identification of dangerous sources of prefabricated buildings and also considering literature research and field investigation, a list of quality risk factors is formed, which constitutes a list of risk factors [24,25]. There are 12 comprehensive risk factors in the risk list, which constitute the second-level indicator factors of the risk evaluation system, which are: production quality, reinforcement quality, laying piping, template quality, transportation process, stacking management, lifting installation, node construction, temporary support, finished product protection, inspection and acceptance, and information. According to the authors [26], the obtained indicator factors are divided into three stages: pre-construction, during-construction, and after construction. Pre-construction mainly refers to the production stage of the prefabrication factory before the precast components are transported to the site. At this stage, the prefabricated components produced by the prefabrication factory are mainly: prefabricated and laminated panels, prefabricated walls, prefabricated stairs, prefabricated beams, and prefabricated pillars. Regarding components stacked to the prefabricated component completion stage, temporary support is the final step of prefabricated component construction. The strength of the nodes can only be removed after construction; the post-construction phase refers to the stage of protection of installed components and consolidation of engineering data. The target layer of the safety evaluation indicator for prefabricated buildings is quality and safety and the constructed evaluation indicator system. According to the evaluation goals and objects, the evaluation system is divided into three layers: the target layer, the first-level indicator layer, and the second-level indicator layer [27]. The target layer is the quality and safety of the prefabricated building, which has the purpose of constructing the evaluation indicators; the criterion layer is the second-level indicator, which mainly evaluates the quality and safety from the three stages of the project’s implementation; the first-level indicator layer is the risk factor that affects the quality and safety issues explicitly.

The quality and safety of prefabricated buildings are the overall target in the evaluation indicator system. Therefore, pre-construction, construction, and post-construction are the first-level indicator layers. Furthermore, production quality, reinforcement quality, laying piping, formwork quality, transportation process, stacking management, hoisting positioning, splicing nodes, temporary support, finished product protection, engineering inspection and acceptance, and integrity and authenticity of engineering data are taken as a secondary indicator layer.

## 2. Materials and Methods

The research methodology relied on a number of hypotheses that are tested by structuring equation modeling. The hypotheses were developed through an in-depth study of previous studies on this subject.

### 2.1. Model Assumption

**H1.**  *Pre-construction significantly impacts prefabricated construction safety risk assessment system*.

**H11.**  *Production quality significantly impacts pre-construction* [12,13,28].

**H12.**  *Reinforcement quality significantly impacts pre-construction* [20,28].

**H13.**  *Laying piping significantly impacts pre-construction* [28].

**H14.**  *Template quality significantly impacts pre-construction* [28].

**H15.**  *Transport process significantly impacts pre-construction* [13,20,28].

**H2.**  *During-construction significantly impacts prefabricated construction safety risk assessment system*.

**H21.**  *Stacking management significantly impacts during-construction* [12,20,28].

**H22.**  *Lifting positioning significantly impacts during-construction* [20,23].

**H23.**  *Splicing node significantly impacts during-construction* [12].

**H24.**  *Temporary support significantly impacts during-construction* [20,28].

**H3.**  *After-construction significantly impacts prefabricated construction safety risk assessment system*.

**H31.**  *Product protection significantly impacts after-construction* [13,23].

**H32.**  *Engineering acceptance significantly impacts after-construction* [28,29,30].

**H33.**  *Completeness and authenticity of engineering data impact after-construction* [29,30].

**Figure 1:**
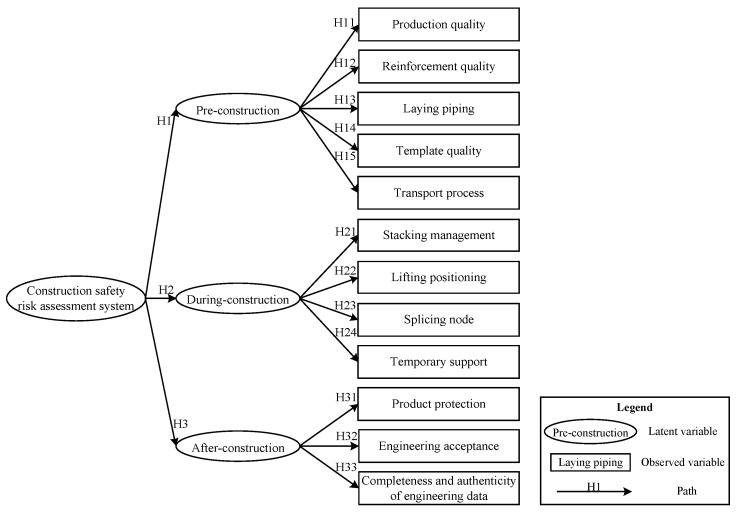
Hypothetical relational model.

The 15 pre-set research hypotheses were combined with the SEM visible module to obtain a hypothetical model. The hypothetical relational model is shown in Figure 1.

### 2.2. Introduction of SEM

SEM is a statistical method to analyze the relationship between variables based on a covariance matrix and is essential for multivariate data analysis. There are two types of variables: the first is the observed variable, which is represented by a square; the second is the latent variable, which is represented by an ellipse. The analysis path includes a measurement model and a structural model. Among them, the calculation equations of measurement model are as follows:

(1) $$X=\Lambda_{X}\xi +\delta$$

(2) $$Y=\Lambda_{Y}\eta +\varepsilon$$

Equation (1) defines the relationship between the explicit observed variable *X* and the implicit observed variable *ξ*; Equation (2) defines the relationship between the explicit latent variable *Y* and the implicit latent variable *η*. *δ* is the residual of the dominant observed variable *X*; *η* is the residual of the dominant latent variable *Y*; Λ*_X_* is the regression coefficient of *ξ*; Λ*_Y_* is the regression coefficient of *η*.

The calculation equation of the structural model is as follows:

(3) η=Bη+Γξ+ζ

Equation (3) represents the relationship between the implicit observed variable *ξ* and the implicit latent variable *η*. *B* is the coefficient matrix between implicit latent variables; *Γ* is the coefficient matrix composed of implicit observed variables and implicit latent variables; ζ is the residual of the structural equation.

Since the model can solve the relationship between multiple causes and multiple results at once, there is no problem with measurement errors for the variables. Furthermore, the degree of fit can be tested after the model is identified. Therefore, this paper selects SEM to study the relationship between the factors affecting the safety risk during the construction phase.

### 2.3. Samples and Data Collection

A questionnaire is an essential form of data collection in this paper. The reliability and validity examinations ensure that the data are scientific. Taking into account the characteristics of the construction industry and the complexity of the research objects, on the premise of ensuring the professionalism of the questionnaire, the design of the items also uses simpler and easy-to-understand words and phrases. The first part uses 5 multiple-choice questions to collect basic information from the research object; the second part collects the research object’s understanding of the importance of the impact indicators through 15 multiple-choice questions; The last part collects the supplementary suggestions of the research object through an open-ended blank-filling question. In order to improve the efficiency of the survey, the questionnaires were issued by a combination of online filling and on-site filling. Questionnaires were distributed to more than 10 typical projects in Fuzhou, Xiamen, and other cities. The main targets for the distribution were prefabricated component industry personnel and construction industry personnel. A total of 450 questionnaires were issued, and 437 were recovered. The recovery rate reached 92.67%, of which the the number of effective questionnaires was 408, and the effective recovery rate was 93.36%, as Table 1.

### 2.4. Introduction of the Reliability and the Validity Examination

**Figure 2:**
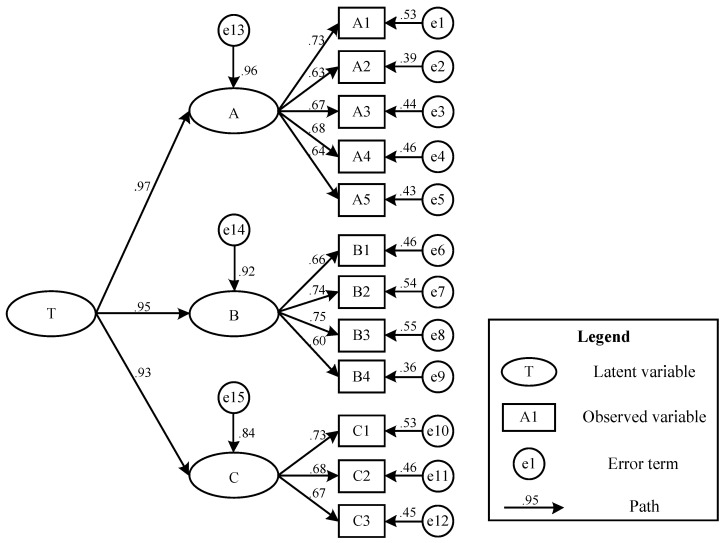
Second-order confirmatory factor analysis structural model diagram.

Reliability is an academic term for the reliability of test results. Generally, internal consistency is used to characterize the reliability of the test. The Cronbach α coefficient (Equation (4)) is a commonly used indicator for reliability analysis. SPSS22.0 software was used for the questionnaire’s reliability test. The Cronbach α coefficient was 0.902, indicating that the test is reliable. Validity is effectiveness, which refers to the accurate measurement degree of the measured object by the measuring tool or means. Effectiveness refers to how the measurement result reflects the content to be inspected. The more consistent the measurement result is with the measured content, the higher the validity; otherwise, the lower the validity—Bartlett sphere testing is used to determine whether the data is suitable for factor analysis. SPSS22.0 software was used for KMO and Bartlett’s test. AMOS22.0 is used to establish a safety risk evaluation system for prefabricated building construction. Moreover, second-order CFA modeling analysis is seen as shown in Figure 2. Since the observed variables will produce errors when estimating latent variables, e represents the residual value. Before evaluating the model, the significance test of the path coefficient and factor load coefficient is carried. The validity of the data can be guaranteed, and the two indicators of C.R and P are generally tested for significance to ensure validity.

### 2.5. Introduction of SDM

System dynamics is an effective method to study complex systems, and most scholars use Vensim software to construct causal circuit diagrams of complex systems. The simulation result obtained by SDM is composed of a horizontal axis representing the step length, a number axis representing the parameter indicator, and a smooth curve of the indicator change. In this paper, the SDM method is used for simulation, which is beneficial to study the relationship and feedback information between system elements as a whole, so as to make up for the limitations of static research and local research [31].

## 3. Results

### 3.1. Reliability and Validity Examination

After the analysis, the KMO value was 0.930, and Bartlett’s test value was significant at *P* = 0. The above indicators indicate that the questionnaire data has a very high correlation, suitable for factor analysis, indicating that the questionnaire meets the validity requirements. The former is used as the main factor, and the cumulative contribution of variance is 58.936%, and more than 50%. This indicates that the scale is validated for validity, and the validity is guaranteed.

### 3.2. Model Building and Identification

There are three latent variables in the model, namely pre-construction, during-construction, and after-construction with, respectively, five observed variables, four observed variables, and three observed variables. Therefore, before performing model analysis, model identification should be performed. In this paper, the *t*-law is used to judge whether the model is recognizable. Where *D_f_* represents the number of measurement data, *t* is the number of variables that need to be observed, and the calculation is as in Equation (4), *t* represents the number of freely estimated parameters; if *D_f_* – *t* > 0, it can be identified; if *D_f_* – *t* < 0, it cannot be identified.

(4) $$D_{f}=\frac{\left(p+q\right)\left(p+q+1\right)}{2}$$

In Equation (4), *p* and *q* are the numbers of exogenous and endogenous observed variables, respectively. Using Equation (4), the number of hypothetical models’ observed data *D_f_* = 18 × 19/2 = 171, *D_f_* − *t* = 171 − 46 > 0 can be analyzed further.

The regression coefficients and standardized regression coefficients in the calculation results in AMOS22.0 can be summarized and observed. The second-order confirmatory factor analysis structural model diagram can be seen in Figure 2.

When all C.R > 2 indicates that the significance level of all observed variables is less than 0.05, the result is significant, and the degree of influence is high. Furthermore, it is known from Table 2 that the value of column P is above 0.001, indicating that these influence levels are significant and that the regression coefficient is not zero. Therefore, it can be concluded that the path coefficients between latent variables are all higher than 0.5, which shows that it is generally suitable, indicating that the influence is enormous and generally meets the requirements of the model. Therefore, the next step of model fitting can be continued. Using AMOS22.0 to calculate the final model, the final result is the total effect of the model. It includes two parts, including direct effect and indirect effect. First, the direct effect is the path coefficient from the cause variable to the result variable. Second, the indirect effect is the sum of the product of the path coefficients of each dependent variable to the result variable, as shown in Table 2.

In Table 2, T represents ”Construction safety risk assessment system”, A represents “pre-construction”, B represents “during-construction”, and C represents “after-construction”; A1 represents “production quality”, A2 represents “reinforcement quality”, A3 represents “laying piping”, A4 represents “template quality”, A5 represents “transport process”, B1 represents “stacking management”, B2 represents “lifting positioning”, B3 represents “splicing node”, B4 represents “temporary support”, C1 represents “product protection”, C2 represents “engineering acceptance”, and C3 represents “completeness and authenticity of engineering data”. It can be seen from Table 2 that in this structural model, the path indices of the three internal latent variables all exceed 0.9. It shows that these three indicators are significant for the prefabricated building construction stage’s risk assessment system and must be considered. Pre-construction factors have the most significant impact. It can be seen that the factory-made quality of assembled components has the most significant impact on construction safety, followed by during-construction and after-construction. Among the indicators of pre-construction, production quality has the most significant impact on the risk assessment system during the construction stage of prefabricated buildings, which shows that production quality has the most significant impact on pre-construction. This shows that managers must always pay attention to the factory quality of prefabricated building components. For laying piping, template quality, reinforcement quality, and transport process, the importance of the pre-construction design and the accessories needed for maintenance should be explained after the component is completed. Attention should be paid to the choice of formwork. For reinforcement, mistakes such as excessive reinforcement damage and minor tendon damage must be avoided.

Furthermore, careful consideration of the choice of vehicle transport mode should be required. The results also show a better solution in the industry for reinforcement and transportation. Finally, they show that these indicators are conducive to avoiding the risk of prefabricated building construction. Sub-hypotheses H11, H12, H13, H14, H15 are all established. Among the various indicators in the during-construction stage, the most significant impact on the risk assessment system during the construction phase of prefabricated buildings is the splicing node, which indicates that the splicing node has the most significant impact on construction. This shows that on-site personnel must pay attention to assembling the building components. Furthermore, the connection of nodes has a significant impact on the stability and seismic performance of the structure. Next, for lifting positioning, it is explained that at this step, the selection of machinery and the control of lifting technology are the most important variables in preventing quality problems caused by machinery and other factors.

For stacking management and temporary support, the results indicate that the supporting management and maintenance of components or structures during construction also have a more significant impact on construction safety. It shows that these indicators are conducive to avoiding the emergence of construction risks. Sub-hypotheses H21, H22, H23, H24 are all established. Among the various indicators of after-construction, the most significant impact on the risk assessment system during the construction phase of prefabricated buildings is product protection, which shows that the protection of finished products has the most significant impact on construction. This shows that the relevant personnel should always pay attention to the protection of the prefabricated building after completing construction. Furthermore, engineering acceptance and the integrity and authenticity of the engineering data guarantee the quality and safety of the structure or components before use and post-maintenance inspection. It also shows that this content in China already has a mature model. Therefore, these indicators are conducive to avoiding the emergence of construction risks. Sub-hypotheses H31, H32, H33 are all established.

### 3.3. Analysis of the Safety Risk System Dynamic Model of Prefabricated Building Construction

#### Causal Loop Analysis

**Figure 3:**
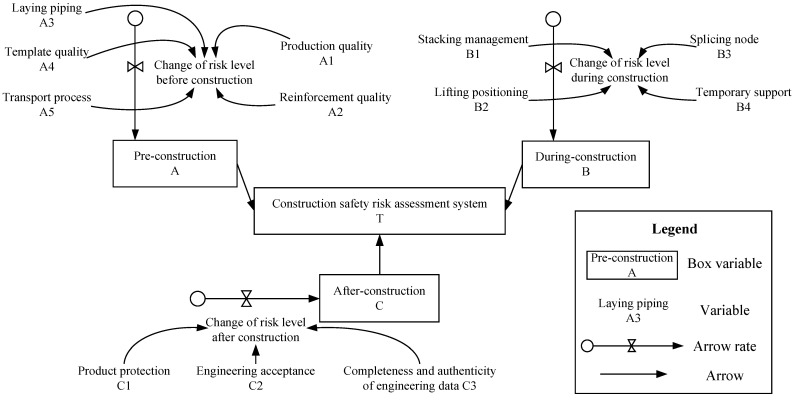
Inventory flow chart of construction safety risk factors.

Causal loop analysis of the safety risk system dynamic model of prefabricated building construction can be seen in Figure 3. It can be seen from the above description that the path coefficients of each observed variable and potential variable have been determined. The system weight of each potential variable is expressed by the ratio of the path coefficient of each potential variable to the sum of the path coefficient of each potential variable. The indicator weight is expressed by the ratio of the corresponding path coefficient and the path coefficient of other observed variables. In the stock flow chart, the values of some variables can be consulted through historical data, while others cannot be determined. To make the findings comparable, all of the numbers are in a range that shows the likelihood of this factor occurring. In this paper, the research on each variable is dimensionless.

Therefore, the collected questionnaire data are reasonably estimated to obtain the initial risk value of the corresponding order of magnitude. The way to determine the value of each variable is shown in Table 3.

### 3.4. Stock Flow Analysis

**Figure 4:**
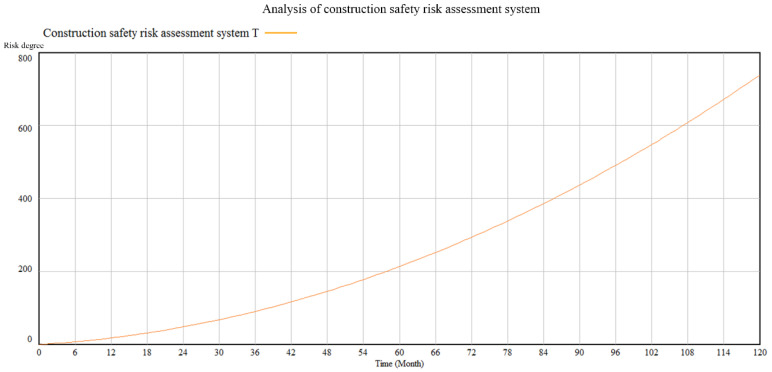
Analysis of construction safety risk assessment system.

**Figure 5:**
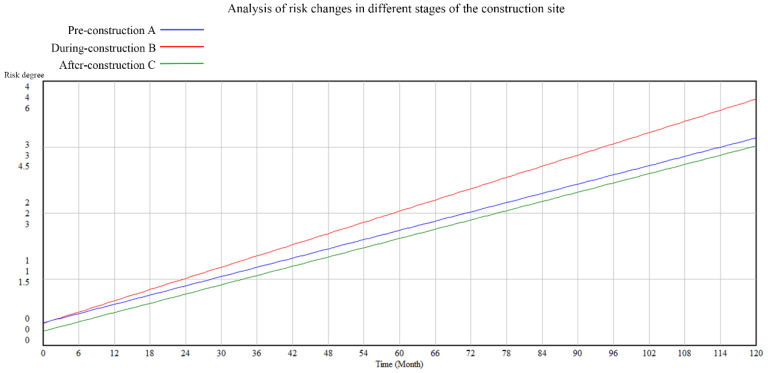
Analy-sis of risk changes in different stages of the construction site.

Assuming that the construction time is 120 months, Vensim software is used for system dynamics analysis and simulation. As a result, the comprehensive risk level of the construction site in a typhoon environment and the risk changes in different stages under the joint action of multiple factors are obtained, as shown in Figure 4 and Figure 5.

It can be seen from the above contents that pre-construction has the most significant influence. The factory quality of prefabricated components has the most significant impact on construction safety, then during-construction and after-construction, which shows that the production quality of the components and parts of the prefabricated building are the first step to ensure safety. Factory preparation is the most significant influencing factor. It also illustrates the technology of prefabricated buildings in China; enough attention should be paid to the deficiencies in the aspects.

## 4. Case Analysis

### 4.1. Basic Information of a Prefabricated Construction Project

There are three prefabricated building projects within the complex residential construction in Changle City, Fujian Province, with a floor area of 40,000 m^2^. Among them, #2 is 34 floors, and the building height is 95.62 m; #20 and #21 buildings are 34 floors, and the building height is 97.07 m. The project’s external walls, stairs, balcony slabs, balcony partitions, and drainage ditches are prefabricated components. The prefabrication rate of the project is 17.2% for #2 and 16.2% for #20 and #21. The project undergoes modular design and unified production in the prefabrication plant and adjusts the production schedule of the prefabrication plant according to the on-site construction progress.

According to the application of multi-level extensions in prefabricated buildings, a prefabricated construction project is used as the evaluation object to establish a multi-level-extension-based quality and safety risk assessment system in Changle City Fujian Province. Pre-construction, during-construction, and after-construction are taken as first-level evaluation indicators, and the lower-level indicators that constitute the first-level indicators are second-level indicators. Examples verify the feasibility and applicability of the method in prefabricated quality management. Thirty experts were invited to participate in the observation of construction workers.

### 4.2. Indicator Weight Calculation and Evaluation

It can be seen from the previous description that the path coefficients of each observed variable and latent variable have been determined. The system weight of each latent variable is represented by the ratio of each path coefficient and the sum of each latent variable path coefficient, and the indicator weight is represented by the ratio of the corresponding path coefficient to the sum of the path coefficients of other observed variables, which can be seen in Table 4.

After the weights of each indicator are calculated, the evaluation method of each part of the model is shown in the following Equation (5):

(5) $$\{\begin{array}{c} R=K_{1}+K_{2}+K_{3}\\ K_{1}=\overset{n=5}{\underset{i=1}{\sum }}A_{i}\times W_{1i}\\ K_{2}=\overset{n=4}{\underset{i=1}{\sum }}B_{i}\times W_{2i}\\ K_{3}=\overset{n=3}{\underset{i=1}{\sum }}C_{i}\times W_{3i} \end{array}$$

**Figure 6:**
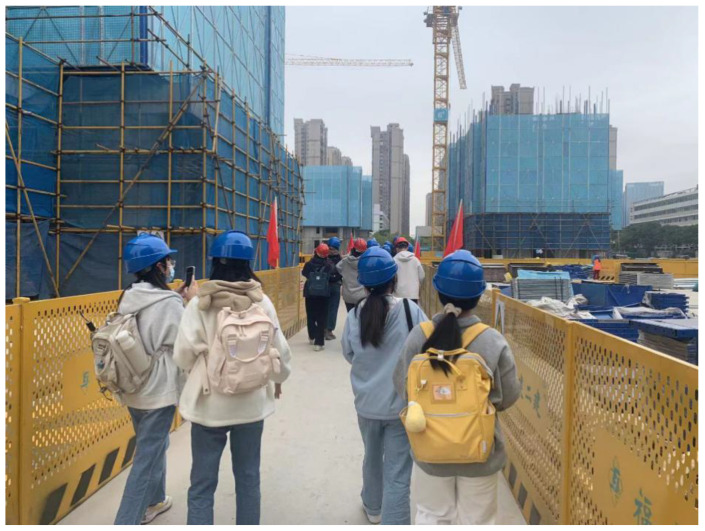
On-site construction pictures of prefabricated building projects.

A prefabricated building construction project in Changle City, Fujian Province, is used as the evaluation object to establish a safety risk evaluation system based on SEM; the project site overview is shown in Figure 6. Taking pre-construction, during-construction, and after-construction as the first-level evaluation indicators, the lower-level indicators that constitute the first-level indicators are the second-level indicators. Examples verify the feasibility and applicability of this method in assembly building construction management. Thirty experts were invited, and selection was based on those engaged in prefabricated construction to sort and sort them out through questionnaires.

Questionnaires were compiled and scored by experts. Then, according to the data in the table, the above calculation formula is used to calculate the system’s score, as shown in Table 5.

It can be seen from Table 5 that the final comprehensive score is 92.71, indicating that the construction safety of the residential building is generally controllable, and the quality is guaranteed.

## 5. Discussion

Based on an example verification of a prefabricated residential construction project in Changle City, the SEM method is used to determine the evaluation indicator weights at all levels, the evaluation matter element and the risk level domain are determined based on the principle of extents, and the correlation function about the risk level is established. Extension evaluation determines the risk level of evaluation indicators at all levels. Corresponding measures based on the evaluation results should be taken.

(1) Pre-construction factors account for the most significant proportion, indicating that the production quality of prefabricated building components is the first step to ensure safety, and factory preparation is the most significant influencing factor. The technical deficiencies of prefabricated buildings are illustrated in China, which should be given adequate attention. From the construction point of view, the splicing node at this stage has the highest score, indicating that the node-splicing construction technology and corresponding organization management are in place, comply with relevant rules and regulations, and produce results safely and reduce risks. On the other hand, the stacking management score is lower than other indicators, and the warehouse management of components needs to be improved. The scores of other indicators are close to that pre-construction, and they also need to be strictly checked. From the point of view of after-construction, the weight of this stage is similar to the previous two stages. Although the weight of the three is the lowest, the general score is higher than the first two. Therefore, it can be considered that the management of the later stages of operation and maintenance has a more developed method. However, the impact on the safety risks during the construction phase is not as high as the former two; that is, although the management model in the later period is constantly improving, it will take time to be accepted by the industry.

(2) Regarding quality and safety management, pre-construction mainly involves prefabricated components’ production and transportation stages. Regarding the reinforcement of components, the problem mainly occurs because the preset position of the reinforcement is unreasonable. The displacement of the reinforcement causes secondary processing at the construction site, which affects the overall structural quality of the component and weakens safety performance. Therefore, the prefabrication plant should strictly check the position of the reinforcement when building concrete and use unique templates to position the rebar. Regarding the quality of component production and component damage during transportation, management should refer to the precautionary measures in [32], strengthen worker management, and strictly control quality; make “L”-shaped plates to reduce the stress of component corners during transportation and aim at large-span components; and reduce the span of transportation and pay attention to the addition of protective blocks between the components to reduce the collision damage caused by bumps in the road. Regarding laying quality, the design pipeline reserve line can be made on the components in advance to prevent installation deviations. When the concrete is poured, the two ends of the pipeline are blocked and protected to prevent concrete from entering the blocked pipeline. For prefabricated components that have been processed, piping and other auxiliary fittings are reinforced to prevent them from falling off. Regarding the quality of the formwork, the formwork is designed with proper support and perimeter spacing according to the specific components. The appropriate material formwork is used for different components. Management should pay attention to the regular maintenance of the formwork and replace the problem formwork promptly. When the release agent is used, it should be uniform and complete.

(3) In terms of quality and safety management in the during-construction stage, the quality problem of splicing nodes is the most challenging point. At present, most of our country uses a sleeve grouting connection. When the mortar flows out of the upper mouth, the upper seal should be blocked promptly. Supervisors should be set up for supervision and inspection, and quality inspection records should be made. The images should be archived, and spot checks should be carried out regularly to enhance construction worker safety education. The fixed area and the flow area should be divided for stacking management regarding the stacking of components. The stacking site should be solid, level, and free of standing water. The stacking time of components should not be too long. The components should be elevated or covered during stacking to reduce damage during component stacking. The number of component-stacking layers should consider the bearing capacity of the stacking site and the strength of the components themselves. Proper safety analysis of the stacking support should be performed. To prevent overturning regarding the hoisting and installation procedures, a quality and safety expert group shall be established to formulate unique, safe construction plans and construction operation specifications, simulate the hoisting plan, and optimize the construction plan; at the same time, a safety commissioner should be set up on-site to guide the construction, and non-standard construction personnel should be strictly prohibited from operating. Communicate the implementation of the project with the construction site promptly and regularly check the condition of the equipment to ensure smooth progress of the lifting work. Regarding temporary support, it is necessary to design safety measures for temporary support equipment to ensure construction safety.

(4) In terms of after-construction quality and safety management, according to the suggestions given in [33,34] regarding the protection of finished products of components, personnel management, strengthening the awareness of quality and safety protection of construction personnel, strengthening the design strength of the components themselves, and strengthening the components are the focuses. Regarding the completeness and truthfulness of inspection and acceptance and engineering data, the construction unit shall establish an engineering inspection and acceptance system and strictly implement it. Project acceptance and construction data should be carried out simultaneously to ensure the authenticity and completeness of the project data. Particular attention should be paid to the inspection and acceptance of concealed projects, and subsequent projects can only be covered after being signed and confirmed by the relevant responsible persons.

Compared with other methods, the combination evaluation meets to fit and modify the evaluation model in the indicator system and improve the accuracy of the evaluation results. Therefore, this study uses a combined evaluation to study the safety risk of prefabricated building construction by establishing a risk evaluation model. The results provide a reference for evaluating safety risk in the construction stages of prefabricated buildings of the same kind.

## 6. Conclusions

Prefabricated buildings have certain advantages over traditional construction, such as improved construction quality, reduced labor force requirements, and lower environmental impact. Therefore, this study explored practical solutions to help reduce the impact caused by using limited resources. To evaluate the states for prefabricated building projects, SEM and SDM were used to analyze the influencing factors of safety risk for prefabricated buildings.

(1) This paper investigates the influencing factors on the safety risk of prefabricated building construction based on a series of surveys in China. Data were collected through a questionnaire survey, and SPSS was used to test the validity and reliability, after which preliminary evaluation indicators were selected. The indicator evaluation system was constructed based on SEM, including three first-level and 12 second-level factors.
(2) SEM and SDM integrate all significant variables affecting the safety risk of prefabricated building projects and can study the behavior of these potential variables from an influencing perspective. These assessments cover risk factors resulting from pre-construction, during-construction, and after-construction risks. Research results show that the most considerable influence was pre-construction, followed by during-construction and after-construction. Stacking management runs through the whole construction process and requires multi-party coordination and management; before construction, the production and transportation of components are the key and difficult points of management, and different protection schemes are prepared for different components; during the construction process, quality inspection records of the splicing joints should be made, and sample inspection should be carried out regularly; in the later stage of construction, reinforcement and protection measures should be strengthened by taking reinforcement and protection measures. Therefore, the suggested solutions provide valuable references for decision-makers to improve the efficiency of the measures.

Therefore, compared with other studies, this study obtained prefabricated building safety risk principal factors by analyzing prefabricated building safety risk during the construction processes, making it more focused on decisionmakers when making relevant measures to reduce the negative impact of prefabricated buildings. Furthermore, this research provides a measurement method and assesses the safety risk of prefabricated building projects as an effective instrument based on integrating SEM and SDM. The result of this study can provide a theoretical reference to control risk management and practical significance for risk reduction policies and regulations for governments and enterprises. Although the objectives of this study were achieved, there are some limitations worth mentioning. First, this study collected the subjective opinions of respondents based on their personal experiences. Second, the findings from this study apply exclusively to the Chinese context and require further investigation to be applied to other countries. Nonetheless, the findings are valuable because they contribute to the body of knowledge on the topic and are the first to investigate the influencing factors of prefabricated building safety risk. Third, the data applied to the analysis were obtained from prefabricated building projects in China, but the research is also helpful for conducting similar studies in other regions.

Furthermore, the findings can benefit decisionmakers in the industry by providing a thorough picture of the safety risk control method in prefabricated building construction, which can ultimately help them achieve greater efficiency when implementing measures to target such risks. Future research should build on this by considering prefabricated building construction safety risks in other contexts. In addition, an accurate risk evaluation for prefabricated building construction should also be established.

## Figures and Tables

**Table 1:** Questionnaire sample data statistics.

Basic Information	Type	Quantity	Proportion
Workplace	State-owned enterprise	199	48.77%
	Private enterprise	96	23.53%
	Foreign enterprise	25	6.13%
	Sino-foreign joint venture enterprise	40	9.80%
	Other enterprises	48	11.76%
Job title	Senior engineer	7	1.72%
	Mid-level engineer	94	23.04%
	Construction technician	203	49.75%
Education level	Student	78	19.12%
	Other	26	6.37%
	PhD	33	8.09%
	Master’s degree	107	26.23%
	Undergraduate degree	224	54.90%
	College degree	32	7.84%
	Other	12	2.94%
Working years	Over 10	2	0.49%
	5–10	30	7.35%
	3–5	131	32.11%
	1–3	150	36.76%
	Intern	95	23.28%
Age	19–22	45	11.03%
	23–30	170	41.67%
	31–40	166	40.69%
	40–50	24	5.88%
	Over 50	3	0.074%

**Table 2:** Final model total effect table.

Factor	Correlation	Factor	Path Coefficient	Sequence
A	<---	T	0.972	1
B	<---	T	0.954	2
C	<---	T	0.925	3
B3	<---	B	0.753	1
B2	<---	B	0.740	2
A1	<---	A	0.733	3
C1	<---	C	0.726	4
C2	<---	C	0.678	5
A4	<---	A	0.676	6
C3	<---	C	0.674	7
A3	<---	A	0.666	8
B1	<---	B	0.662	9
A5	<---	A	0.638	10
A2	<---	A	0.629	11
B4	<---	B	0.600	12

**Table 3:** Variable value and expression.

Variable Type	Serial Number	Risk Value
Observed variables	A1	0.0750
	A2	0.0641
	A3	0.0679
	A4	0.0689
	A5	0.0651
	B1	0.0804
	B2	0.0901
	B3	0.0915
	B4	0.0730
	C1	0.1131
	C2	0.1056
	C3	0.1053
Latent variables	A	INTEG (change of risk level before-construction, 0.341)
	B	INTEG (change of risk level during-construction, 0.335)
	C	INTEG (change of risk level after-construction, 0.324)

**Table 4:** Model effect.

Latent Variable $\left(F_{n}\right)$	System Weight $\left(W_{F_{n}}\right)$	Measurement Variable $\left(I_{nm}\right)$	Indicator Weight $\left(W_{I_{nm}}\right)$	Indicator Total Weight $\left(W_{nm}=F_{n}\times W_{I_{nm}}\right)$
Pre-construction $\left(F_{1}\right)$	$0.341\;(W_{F_{1}})$	Production quality $\left(I_{11}\right)$	0.220	0.0750
		Reinforcement quality $\left(I_{12}\right)$	0.188	0.0641
		Laying piping $\left(I_{13}\right)$	0.199	0.0679
		Template quality $\left(I_{14}\right)$	0.202	0.0689
		Transport process $\left(I_{15}\right)$	0.191	0.0651
During-construction $\left(F_{2}\right)$	$0.335\;(W_{F_{2}})$	Stacking management $\left(I_{21}\right)$	0.240	0.0804
		Lifting positioning $\left(I_{22}\right)$	0.269	0.0901
		Splicing node $\left(I_{23}\right)$	0.273	0.0915
		Temporary support $\left(I_{24}\right)$	0.218	0.0730
After-construction $\left(F_{3}\right)$	$0.324\;(W_{F_{3}})$	Product protection $\left(I_{31}\right)$	0.349	0.1131
		Engineering acceptance $\left(I_{32}\right)$	0.326	0.1056
		Completeness and authenticity of engineering data $\left(I_{33}\right)$	0.325	0.1053

**Table 5:** Indicator scores.

First-Level Indicators		Second-Level Indicators	Indicator Weight	Scores	Final Scores
		Production quality	0.0750	92	6.90
		Reinforcement quality	0.0641	96	6.15
Pre-construction	31.5	Laying piping Template quality	0.0679 0.0689	91 93	6.18 6.41
		Transport process	0.0651	90	5.86
		Stacking management	0.0804	89	7.16
		Lifting positioning	0.0901	92	8.29
During-construction	31.02	Splicing node	0.0915	96	8.78
		Temporary support	0.0730	93	6.79
		Product protection	0.1131	91	10.29
After-construction	30.19	Engineering acceptance	0.1056	94	9.93
		Completeness and authenticity of engineering data	0.1053	93	9.79
Total	92.71	-	1	-	92.71

## Data Availability

The data sets during and/or analyzed during the current study are available from the corresponding author on reasonable request.
